# The cognitive impairment and risk factors of the older people living in high fluorosis areas: DKK1 need attention

**DOI:** 10.1186/s12889-021-12310-6

**Published:** 2021-12-09

**Authors:** Chao Ren, Peng Zhang, Xiao-Yan Yao, Hui-Hua Li, Rui Chen, Cai-Yi Zhang, De-Qin Geng

**Affiliations:** 1 Department of Neurology, The Affiliated Hospital of Xuzhou Medical University, Xuzhou, 221006 China; 2 Department of Neurology , Department of Neurology Yantai Yuhuangding Hospitalof Qingdao University, Yantai, 264000 China; 3 Department of Psychiatry and Psychology, The Affiliated Xuzhou Oriental Hospital of Xuzhou Medical University, Xuzhou, 221000 China; 4 Zhenjiang Mental Health Center, The Fifth People’s Hospital of Zhenjiang City, Zhenjiang, 212000 China; 5 Department of Neurology, The Affiliated Huai’an Hospital of Xuzhou Medical University, Huai’an, 223002 China

**Keywords:** Cognition, DKK1, Fluoride, Drinking water, Risk factors

## Abstract

**Objective:**

To evaluate cognitive impairment and risk factors of elders in high fluoride drinking water areas and investigate whether DKK1 is involved in this disorder.

**Methods:**

MoCA-B and AD-8 were used to measure the cognitive functions of 272 and 172 subjects over the age of 60 came from the high and normal fluoride drinking water areas respectively, general information and peripheral blood were collected, the level of SOD, GSH and MDA were measured, mRNA level of DKK1, the concentration of blood fluoride and the polymorphism of APOE were tested.

**Results:**

The blood fluoride concentration, mRNA level of DKK1 and ratio of abnormal cognitive function of subjects in high fluorine drinking water areas were higher than those in normal areas. The level of SOD of subjects in high fluorine drinking water was low compared with those in normal areas. The level of MDA and GSH had no difference between the two crowds in different fluorine drinking water areas. There were differences in cigarette smoking, education, dental status, hypertension, hyperlipidaemia and APOE results between the two crowds in different fluorine drinking water areas. The mRNA level of DKK1 and the level of cognitive function showed a positive correlation and DKK1 was one of five risk factors involved in cognitive impairment of older people living in high fluorosis areas.

**Conclusions:**

The cognitive functions could be impaired in the older people living in high fluoride drinking water areas, and DKK1 may as a potential intervention point of this brain damage process need attention.

## Introduction

Fluoride can easily be found in our daily life, and humans may suffer from damage induced by fluorosis [1–3]. Some reports have suggested that excessive fluoride can cause impairments in many systems of the human body. Dental fluorosis and bone damage can easily occur. However, it takes a longer time for side effects related to fluoride to manifest in the central nervous system. Fluoride exposure can affect emotions and cognition in animal models and in children during development [4–6]. One study reported that the intelligence quotient (IQ) and executive functions were lower in individuals living in high fluoride drinking water areas [7]. In our previous study, we found that cognitive disorders resulted from drinking water with high fluoride levels in rat model [8]. The specific mechanism of fluorosis in cognitive disorders was unclear, but some studies showed that oxidative stress damages, neuron apoptosis, changes in neurotransmitters, and synaptic dysfunctions are involved [9–11]. It suggested that oxidative stress damages deserve attention, for the fragmentation and redistribution of mitochondria and the imbalance between the mitochondrial fusion and fission were found in the neurons of the rat exposure to chronic fluoride. Under this circumstance, the respiratory electron transport chain was disturbed and more superoxide radicals were produced. These changes will result in the high level of oxidative stress and contribute to the brain injury [12]. For instance, we found that the antioxidant substance such as superoxide dismutase (SOD) and glutathione (GSH) decreased significantly in rat model of fluorosis in our previous study [8]. It indicated the linkage between cognitive impairment induced by fluoride and oxidative stress.

Recent studies have indicated that cognitive dysfunction is related to many different cell signaling pathways. One study reported that the canonical Wnt signalling pathway was involved in the development of Alzheimer’s disease (AD) [13]. The Wnt signalling pathway plays an important role in neurogenesis, neuroplasticity, memory and learning, which may be potential mechanisms for cognitive impairments [14–16]. Dickkopf-1 (DKK1), a canonical Wnt signalling pathway inhibitor, may bind to and sequester LRP5/6 and then disrupt Wnt- Frizzled-LRP6 complex formation, which would cause changes in down-stream pathway activities [17]. Our research team have recently demonstrated that the canonical Wnt pathway was involved in the fluoride-induced impairment of PC-12 cells [18] and BV2 cells [19], and we found the expression level of DKK1 was significantly higher in the fluoride group than that in control group. Excitingly, more and more studies proved DKK1 expression level increased significantly in the cerebrospinal fluid, plasma and brain tissue of AD patients and AD transgenic mice [20, 21]. In our recent review, we supposed DKK1 may be a key mediator and potential risk factor for AD development, and it may also be as a novel intervention point of brain damage prevention that need attention [22].

Some reports suggested the cognition was impaired in high fluoride drinking water areas [23, 24]. However, the difference of risk factors of cognitive impairment between induced by fluorosis and AD in older people were not clear, not to speak of whether DKK1 is involved in this disorder (cognitive impairment induced by fluoride). Based on these, we investigated the cognitive level of older people and the related risk factors in the high fluoride drinking water areas in China. In brief, our aim was to investigate the risk factors for cognitive disorders induced by high fluoride drinking water and the relationship between cognitive function and the expression of DKK1.

## Methods and materials

### Study design and sample size

The study was carried out with an observational cross-sectional survey design and performed from 1-Jan-2016 to 28-Feb-2017. The sample size was calculated by the PASS software. The calculation formula is below:

$$\mathrm{n}=\frac{2\overline{p}\ \overline{q}{\left({Z}_{\alpha }+{Z}_{\beta}\right)}^2}{{\left(P1-P2\right)}^{2.}}$$

P1 = 0.2, P2 = 0.4, α = 0.05, Z_0.05_ = 1.96, β = 0.9, Z_β_ = 1.28, n_min_ = 110.

### Study of subjects

The fluoride concentration of drinking water was high in the Feng County, Xuzhou City, Jiangsu Province, China, where dental fluorosis was very common. However, there was no data available regarding the cognitive function of the individuals who lived in this area. In this study, a total of 272 subjects from the high fluoride drinking water community (water fluoride concentration > 2 mg/L) of Feng County, Xuzhou City, Jiangsu Province, China, were randomly enrolled in this study. And a total of 172 subjects, from the normal fluoride drinking water community (water fluoride concentration < 0.8 mg/L) of Suining County, Xuzhou City, Jiangsu Province, China, were randomly chosen as the control group. These two counties have a similar culture, lifestyle and economic development level. Individuals with cerebral ischaemia, brain tumours and psychiatric disorders were excluded. This study was approved by the Ethics Committee of Affiliated Xuzhou Oriental Hospital of Xuzhou Medical University, China.

### General information

Socio-demographic and personal information was collected from each subject. These data included gender, age, education, alcohol drinking, smoking, dental status, hypertension, hyperlipidaemia, diabetes and family history (psychiatric diseases or dementia). Age was categorized into 3 groups as follows: 60–69 years of age, 70–80 years of age and older than 80 years of age. Education was categorized into the following groups: illiterate, primary school, middle school, and high school or higher. Dental status was categorized as follows: dental fluorosis, normal and dentures.

### Cognitive function tests

The Montreal Cognitive Assessment-Basic (MoCA-B) and AD-8 were used to investigate the cognitive functions of the subjects. The MoCA-B had excellent validity in screening for mild cognitive impairment in poorly educated older adults regardless of literacy [25]. The AD-8 is an 8-item informant-based questionnaire, which was designed to detect changes in the fields of memory, orientation, judgement and executive function [26].

### Blood sampling and pretreatment

A total of 4 tubes of venous blood (5 ml each) were collected and centrifuged at 5000 rpm/min for 5 min. Sera were stored at − 80 °C. These samples were used for testing the mRNA level of DKK1, fluoride concentration, SOD, GSH, malondialdehyde (MDA) concentration and apolipoprotein E (APOE) gene polymorphism.

### Biochemical tests

Blood fluoride concentration was measured using the method of fluoride ion selective electrode method. Briefly, different concentrations of Fluoride Standard Liquid reagent were used to make a standard line, and then the concentration of the blood samples was adjusted according to the standard line.

The different oxidative stress status was evaluated by measuring levels of SOD, GSH and MDA according to the manufacturer’s instructions in the reagent kit (Nanjing, Jiancheng, China).

The mRNA level of DKK1 was measured using qRT-PCR. Briefly, total human blood RNA was isolated with Trizol reagent (ProbeGene, China), and the concentration was measured using ultraviolet spectrophotometry. Reverse transcription was achieved using the cDNA Synthesis Kit (ProbeGene, China), and qRT-PCR amplification was performed using the SYBR-Green Master mix (Probegene MQ051, China) with the following amplification conditions: 95 °C for 10 min, 40 cycles of 95 °C for 15 s, 60 °C for 30 s, and 72 °C for 2 min. The amplification primer sequences were F: 5′- TCA TAG CAC CTT GGA TGG GTA TTC - 3′, and R: 5′- TTG GAC CAG AAG TGT CTA GCA CAA - 3′. The results were analysed using the ABI2720 PCR System (Applied Bio-systems, USA).

For analysis of APOE gene polymorphisms (also known as genotype), genomic DNA was extracted from collected venous blood samples using a commercial kit (QIAamp DNA Blood Mini Kit, Qiagen, Shanghai, China) beforehand. Then, the potentially mutated positions in 112 (rs429358) and 158 (rs7412) of the APOE gene were conducted by a detection kit (GeneChip Assay, Sinochip, Zhuhai, China) based on genomic DNA. First, all samples were amplified with the Verti™ DX Thermal Cycler (Life Technologies, Singapore) (45 cycles, 94 °C for 30 s and 65 °C for 45 s); then the amplified products were assayed by the fully automated GeneChip detection system (Sinochip, Zhuhai, China). All genotyping results were obtained from the GeneChip automated analysis system.

### Statistical analysis

Socio-demographic factors and numeration data were analysed using the chi-square (χ^2^) test. The nonparametric rank sum test was used to compare the quantitative data between two groups. The degree of association between fluoride-induced cognitive impairment and risk factors was analysed using binary logistic regression analysis. Bivariate correlation analysis was performed using Spearman correlation analysis. SSPS16.0 software was used to analyse all data. A *p*-level of 0.05 was considered statistically significant.

## Results

### Demographic characteristics of subjects in the normal fluoride group and high fluoride group

The demographic characteristics of subjects are displayed in Table 1. The majority of subjects are female (*N* = 119, 69.2% VS *N* = 166, 61% in the two areas, respectively). Over 70% (*N* = 122) of the subjects did not receive education in normal drinking area. The rate of fluorosis teeth (*N* = 266, 97%), hypertension (*N* = 103, 37.7%) and hyperlipemia (*N* = 34,12.5%) are higher in the participants who lived in the high fluoride drinking area than these who lived in the normal fluoride drinking area.

**Table 1:** The socio-demographic characteristics of subjects

	Normal Fluoride Group	High Fluoride Group	X^2^	*p*
**General information**				
Gender	172	272	3.050	0.081
Male	53(30.8%)	106(39%)		
Female	119(69.2%)	166(61%)		
Age (years)			1.118	0.572
60–69	84(48.8%)	132(48.5%)		
70–79	73(42.4%)	108(39.7%)		
≥ 80	15(8.7%)	32(11.8%)		
Education			24.765	0.000^*^
Illiterate	122(70.9%)	137(50.4%)		
Primary school	31(18%)	88(32.4%)		
Middle school	13(7.6%)	44(16.2%)		
High Middle school and higher	6(3.5%)	3(1.1%)		
**Risk factors**				
Cigarette smoking			6.938	0.008^**^
Yes	14(8.1%)	46(16.9%)		
No	158(91.9%)	226(83.1%)		
Alcohol consumption			–	0.43
Yes	0(0%)	1(0.4%)		
No	172(100%)	271(99.6%)		
**Chronic diseases**				
Hypertension			4.658	0.031^*^
Yes	48(27.9%)	103(37.9%)		
No	124(72.1%)	169(62.1%)		
Hyperlipemia			10.439	0.001^*^
Yes	6(3.5%)	34(12.5%)		
No	166(96.5%)	238(87.5%)		
Hyperglycemia			0.092	0.761
Yes	14(8.1%)	20(7.4%)		
No	158(91.9%)	252(92.6%)		
**APOE gene polymorphisms**				
Genotype			71.753	0.000^**^
E2/E2	1(0.6%)	1(0.4%)		
E2/E3	28(16.3%)	139(51.5%)		
E2/E4	0(0%))	4(1.5%)		
E3/E3	143(83.1%)	123(45.2%)		
E3/E4	0(0%)	5(1.8%)		
**Physical sign of fluorosis**				
Teeth			476.841	0.000^*^
Normal	155(90.1%)	2(0.7%)		
Dental fluorosis	9(5.2%)	266(97.8%)		
Denture	8(4.7%)	4(1.5%)		

*APOE* apolipoprotein E

^*^ *P* < 0.05

^**^ *P* < 0.01

### The blood fluoride concentration in subjects from normal and high fluoride drinking water areas

**Fig. 1:**
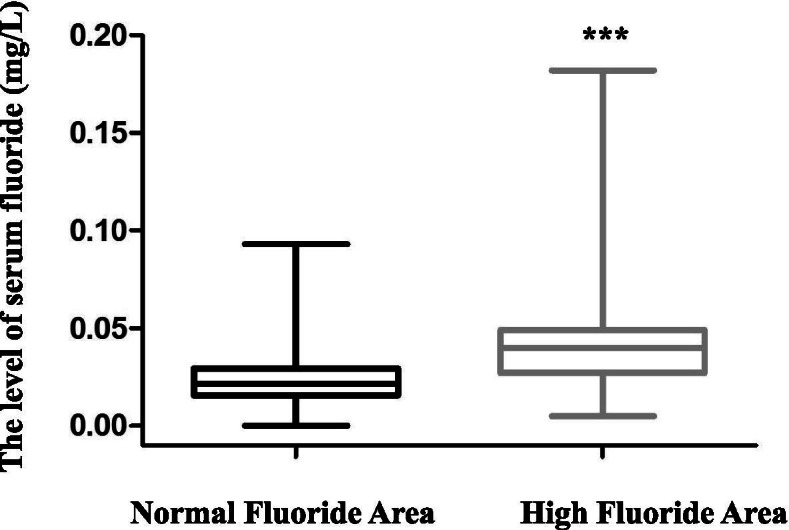
The blood fluoride concentration in subjects from high and normal fluoride drinking water areas is shown. The blood fluoride concentration was higher in subjects form the high fluoride drinking water areas than those from normal fluoride drinking water areas. ***P < 0.001

The blood fluoride concentration of subjects was higher in the high fluoride drinking water areas [0.04 (0.027–0.049) mg/L] compared with the fluoride concentration in subjects from the normal fluoride drinking water areas [0.02 (0.016–0.029) mg/L] (Fig. 1).

### Associations between fluoride and oxidative stress

**Fig. 2:**
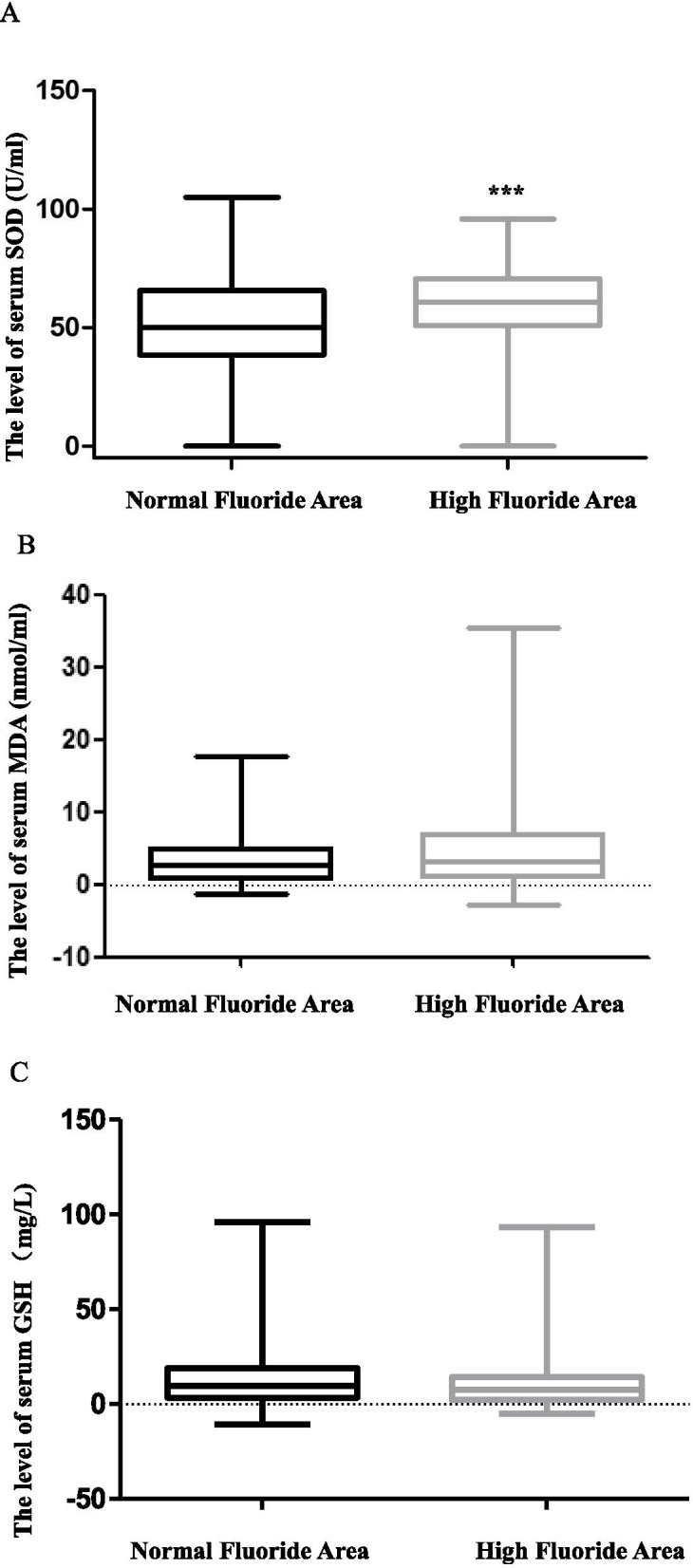
The level of SOD, MDA and GSH in subjects from high and normal fluoride drinking water areas: **A** The level of SOD, **B** The level of MDA, **C** The level of GSH. The level of SOD decreased significantly in the high fluoride drinking water areas. The level of MDA and GSH had no significant difference between the high and normal fluoride water drinking areas. ***P < 0.001. GSH: glutathione, MDA: malondialdehyde, SOD: superoxide dismutase

The level of SOD, an anti-oxidative factor, increased significantly in subjects from the high fluoride water drinking areas [60.66(50.73–70.62)U/mL] (Fig. 2A). The level of MDA, a pro-oxidative factor, and GSH, another anti-oxidative factor had no significant difference between the high and normal fluoride water drinking areas (Fig. 2B, C).

### Fluoride increased the mRNA level of DKK1

**Fig. 3:**
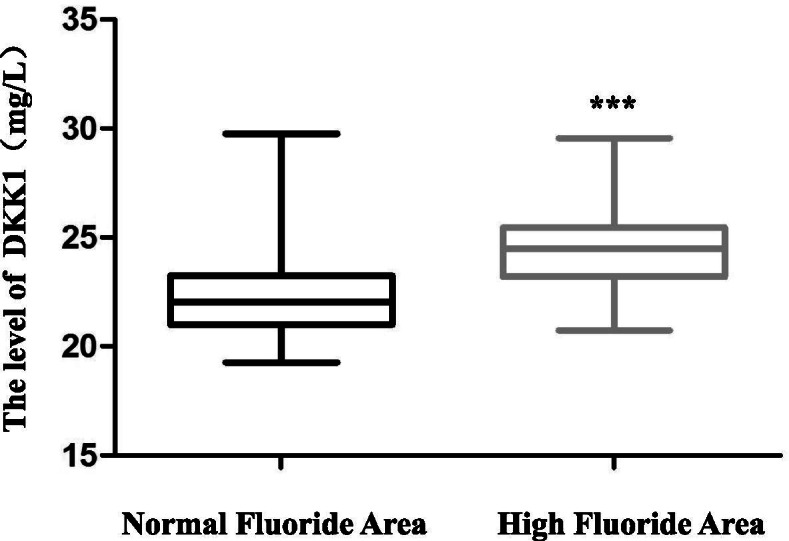
The mRNA level of DKK1 in the high and low fluoride drinking water groups is shown. The mRNA level of DKK1 was higher in subjects form the high fluoride drinking water areas than those from normal fluoride drinking water areas. ***P < 0.001. DKK1: dickkopf-1

DKK1 is an inhibitor of the canonical Wnt signalling pathway and is often associated with different diseases. Our previous studies (cellular and animal models) indicated that there was some relationship between DKK1 and fluoride. In this study, we measured the mRNA level of DKK1 by qRT-PCR. The mRNA level of DKK1 was significantly higher in subjects form the high fluoride drinking water areas [24.47(23.19–25.47)] than those from normal fluoride drinking water areas [22.05(20.99–23.24)] (Fig. 3).

### The incidence of cognitive impairment in older people increased in high fluoride drinking water areas

The MoCA-B and AD-8 were used to measure the cognitive function of subjects. The results suggested that the ratio of abnormal cognitive function of study population in high fluorine drinking water areas were higher (*N* = 124, 45.6%)than those (*N* = 26, 15.1%) in normal areas **(**Table 2**)**.

**Table 2:** Statistical analysis of the incidence of cognitive impairment measured by the AD-8 and MoCA-B

		AD-8/MoCA-B			
		Normal	Abnormal	Total	
Group	Normal	146 (84.9%)	26 (15.1%)	172(100%)	
					X^2^ = 43.736
	Fluoride	148(54.4%)	124(45.6%)	272(100%)	*P* = 0.000^**^
Total		294(66.2%)	150(33.8%)	444(100%)	

*AD-8* Alzheimer’s Disease-8, *MoCA-B* Montreal Cognitive Assessment-Basic

^**^ *P* < 0.01

### The effect of education on cognitive function in subjects from different fluoride drinking water areas

We investigated the effects of different levels of education on cognitive function in both groups. The incidence of cognitive impairment was higher in study population with illiterate (*N* = 52, 38%) and primary school education level (*N* = 47, 53.4%) who lived in the high fluoride drinking water area compared with those (illiterate *N* = 12, 9.8%, primary school *N* = 7, 22.6%)from normal fluoride drinking water areas. However, the incidence of cognitive impairment in the study population with middle school (*N* = 4, 30.8%; *N* = 24, 54.5%) or high and over high middle school (*N* = 3, 50%; *N* = 1, 33.%) education level was not significantly different between the two groups **(**Table 3**)**.

**Table 3:** The effect of level of education on cognitive impairment in subjects from different fluoride drinking water areas

Education	AD-8/MoCA-B		
	Normal	Abnormal	Statistical indicators
Illiterate			
Control Group	110(90.2%)	12(9.8%)	X^2^ = 27.428
Fluoride Group	85(62%)	52(38%)	*P* = 0.000^**^
Primary school			
Control Group	24(77.4%)	7(22.6%)	X^2^ = 8.79
Fluoride Group	41(46.6%)	47(53.4%)	*P* = 0.003^**^
Middle school			
Control Group	9(69.2%)	4(30.8%)	X^2^ = 1.418
Fluoride Group	20(45.5%)	24(54.5%)	*P* = 0.234
High Middle school and higher			
Control Group	3(50%)	3(50%)	–
Fluoride Group	2(66.7%)	1(33.3%)	*P* = 1.000

*AD-8* Alzheimer’s Disease-8, *MoCA-B* Montreal Cognitive Assessment-Basic

^**^ *P* < 0.01

### Correlation coefficient

In this study, spearman correlation analysis was used to investigate the relationship between some of observed variables (fluoride, DKK1and MoCA-B). The result was showed in the Table 4. There was a positive correlation (*r* = 0.313) between the level of DKK1 and the concentration of fluoride(*r* = 0.313, *p* < 0.05), and MoCA-B (*r* = 0.320, *p* < 0.05).

**Table 4:** A correlation of matrix of DKK1, blood concentration of Fluoride and MoCA-B

Variables	Fluroide	DKK1	MoCA-B
Fluroide	1		
DKK1	0.313	1	
MoCA-B	0.320	0.365	1

*DKK1* dickkopf-1, *MoCA-B* Montreal Cognitive Assessment-Basic

### Risk factors

Risk factors of cognitive impairment were analysed using binary logistic regression analysis. As showed in Table 5, age, education, fluoride, DKK1 and dental fluorosis may be the risk factors of cognitive impairment in older people who lived in high fluorosis areas.

**Table 5:** The risk factors of cognitive impairment were analyzed by regression logistics analysis

Variables in the Equation								
	B	S.E.	Wald	df	Sig.	Exp(B)	95.0% C.I.for EXP(B)	
							Lower	Upper
Step 1a								
Group	0.646	0.677	0.91	1	0.34	1.908	0.506	7.188
Gender	0.381	0.376	1.026	1	0.311	1.464	0.7	3.06
Age			14.678	2	0.001			
Age (1)	0.765	0.264	8.42	1	0.004	2.15	1.282	3.606
Age (2)	1.41	0.417	11.432	1	0.001	4.094	1.808	9.268
Education			16.02	3	0.001			
Education (1)	1.035	0.325	10.145	1	0.001	2.815	1.489	5.322
Education (2)	1.405	0.431	10.618	1	0.001	4.076	1.751	9.49
Education (3)	2.015	0.884	5.202	1	0.023	7.502	1.328	42.389
Height	−0.004	0.022	0.037	1	0.848	0.996	0.954	1.039
Weight	−0.008	0.016	0.254	1	0.614	0.992	0.962	1.023
Fluoride	20.047	6.604	9.215	1	0.002	5.08E+ 08	1.22E+ 03	2.12E+ 14
DKK1	0.318	0.081	15.323	1	0.000	1.374	1.172	1.611
Cigarette smoking	0.132	0.391	0.113	1	0.736	1.141	0.53	2.455
Alcohol consumption	−20.128	4.02E+ 04	0.000	1	1	0.000	0.000	
Dental fluorosis			7.896	2	0.019			
Fluorosis (1)	1.734	0.687	6.365	1	0.012	5.665	1.472	21.792
Fluorosis (2)	1.683	0.787	4.58	1	0.032	5.384	1.152	25.153
Hypertension	−0.04	0.266	0.022	1	0.881	0.961	0.57	1.619
Hyperglycemia	0.118	0.476	0.062	1	0.804	1.125	0.443	2.858
Hyperlipemia	−0.379	0.425	0.795	1	0.373	0.684	0.297	1.575
SOD	−0.009	0.008	1.422	1	0.233	0.991	0.975	1.006
GSH	0.004	0.009	0.157	1	0.692	1.004	0.985	1.022
MDA	0.014	0.025	0.343	1	0.558	1.014	0.967	1.065
APOE			0.491	4	0.974			
APOE(1)	20.42	2.67E+ 04	0	1	0.999	7.38E+ 08	0	
APOE(2)	20.36	2.67E+ 04	0	1	0.999	6.96E+ 08	0	
APOE(3)	20.499	2.67E+ 04	0	1	0.999	7.99E+ 08	0	
APOE(4)	21.105	2.67E+ 04	0	1	0.999	1.47E+ 09	0	
Constant	8.132	4.06E+ 04	0	1	1	3.40E+ 03		

Notes

1. The age variables in this study are calculated in comparison with the “60–69 years old” group. The parameter of the Age (1) row gives the OR value and *P* value to the “70–79 years old” group relative to “60–69 years old” group. The parameter of the Age (2) row gives the OR value and *P* value to the “80 years old and above” group relative to “60–69 years old” group

2. The variables of education level in this study are calculated in comparison with the “illiterate” group. The parameter of the Education (1) row gives the OR value and *P* value of the “primary school” group relative to “illiterate” group. The parameter of the Education (2) row gives the OR value and *P* value to the “junior high school” group relative to “illiterate” group. The parameter of the Education (3) row gives the OR value and *P* value to the “high school and above” group relative to “illiterate” group

3. The variables of dental fluorosis in this study are calculated in comparison with the “normal teeth” group. The parameter of the Fluorosis (1) row gives the OR value and *P* value of the “dental fluorosis” group relative to “normal teeth” group. The parameter of the Fluorosis (2) row gives the OR value and P value to the “all dentures (no teeth or false braces)” group relative to “normal teeth” group

4. The APOE variables in this study are calculated in comparison with “E2/E2” group. The parameter in APOE (1) row gives the OR value and P value to “E2/E3” group relative to “E2/E2” group”. The parameter in APOE (2) row gives the OR value and P value to the “E2/E4” group relative to “E2/E2” group. The parameter in APOE (3) row gives the OR value and P value to the “E3/E3” group relative to “E2/E2” group. The parameter in APOE (4) row gives the OR value and P value to the “E3/E4” group relative to “E2/E2” group

*APOE* apolipoprotein E, *DKK1* dickkopf-1, *GSH* glutathione, *MDA* malondialdehyde, *SOD* superoxide dismutase

## Discussion

Fluoride is a necessary element for the development and growth of organs in the human body, and it is often found in our environment [27, 28]. Frequently fluoride is added to toothpaste for the prevention of tooth decay. Since the 1980s, the use of fluoride in dental products has increased significantly. Furthermore, drinking water is a main source of fluoride, and the concentration of fluoride ranges from 0.1–0.8 mg/L [29]. However, the safety margin of fluoride is very narrow. Therefore, it is easy to suffer from fluorosis in high fluoride drinking water areas. The problems of high fluoride drinking water associated with dental and bone fluorosis had frequently been reported. Recently, some reports suggested that the excessive intake of fluoride could induce cognitive impairment in a rat model and in individuals who live in high fluoride drinking water areas [24]. Children who were exposed to high fluoride drinking water in China and India showed decreases in IQ [5, 30].

General information is shown in Table 1. As shown in Table 1, differences in the level of education, hypertension, and APOE polymorphism were observed between the fluoride group and the control group. The blood fluoride concentration was tested and found to be higher in subjects from high drinking water areas than in subjects from normal drinking water areas, as shown in Fig. 1. However, toxic blood fluoride concentrations were not detected, which means that even in high fluoride drinking water areas, the blood fluoride concentrations in older people were considered safe. That may be the reason that the cognitive impairment induced by fluoride was not serious. Since a previous study showed that fluoride (700 μmol/L NaF) could stimulate the ability of cellular anti- oxidative effects [31]. Therefore, it could be inferred that a certain low dose of fluoride exposure may play a protective rather than adverse role on cognitive impairment through the mechanisms of stimulating cells viability and anti-oxidative ability [24]. Moreover, fluorosis was associated with some other factors including age, kidney function, and sex [32]. The results showed that the incidence of dentures was higher in subjects in the normal fluoride drinking water areas. Fluoride is often added to toothpaste for the prevention of cavities, although the absence of fluoride does not necessarily contribute to the development of cavities. Nevertheless, fluoride had a protective effect against damage to the teeth caused by acid.

Oxidative stress is very common in patients with cognitive impairment, as usual, anti-oxidative factors decreased and pro-oxidative factors increased significantly. In our previous study, damage associated with oxidative stress from fluorosis was observed in a rat model [8]. In the present study, we measured two factors related to the oxidative stress. The results indicated that the level of SOD increased significantly in subjects from high fluoride drinking water areas. There was no significant difference in the level of GSH and MDA between the two groups. This result was not completely consistent with the results of our previous animal experiment [8]. The reason may be that the higher concentration of fluoride (not a toxic concentration) stimulated the activation of the oxidative system, which led to an increase in the level of the anti-oxidative stress factor SOD. These findings could indicate that a protective and reactive mechanism was engaged in response to the increased blood fluoride concentration [31]. What’s more, it further supported the viewpoint presented by Li et al. that the certain low doses of fluoride intake may be a potential protective rather than a harmful factor for cognitive function; however, high fluoride exposure is a potential risk factor for cognitive impairment in older population [24].

As shown in Tables 1 and 3, although the prevalence of subjects who were illiterate was lower in the high fluoride drinking water areas, the incidence of abnormal cognitive function was higher. This result suggested that fluoride could increase the incidence of cognitive impairment. Additionally, we found that less education was associated with a higher incidence of cognitive impairment. For subjects who received more than 8 years of education (above middle school in China), no significant difference in cognitive function was observed between subjects from high fluoride drinking water areas and those from normal fluoride drinking water areas. This finding possibly suggested that the severity of cognitive impairment induced by fluoride was less in person with the higher level of education. More and more researches suggest that people with more education have lower prevalence of dementia [33]. Some scholars believe that this is benefit from the high cognitive reserve in individuals with high education [34]. But this protective effect of a high-education level may only be stronger in the early stage of disease [35]. The older persons with cognitive impairment induced by fluorosis in our study may be in the early stages of the disease. Meanwhile, other studies have found that high education may offer protection against tauopathy in patients with mild cognitive impairment [36].

DKK1, an inhibitor of the canonical Wnt signalling pathway, has been reported to be involved in cognitive impairment [37]. Increased DKK1 is associated with AD [38]. A positive correlation exists between cognitive function and DKK1 expression in diabetic rats, and there is also a positive correlation between DKK1-reducing therapy and improved cognitive function in rats [39]. Our recent research found that the level of DKK1 increased significantly in PC-12 cells [18] and BV2 cells [19] exposed to fluoride. However, the expression level of DKK1 in older people from high fluoride drinking water areas is unknown. The mRNA level of DKK1 was measured in both groups. The level of DKK1 increased significantly in subjects from high fluoride drinking water areas compared with that in subjects from normal fluoride drinking water areas. And we also investigated the correlation between observed variability. The result implied that DKK1was correlated with cognition and Fluoride concentration, although the correlation was weak. This finding suggested that DKK1 could be an indicator of cognitive impairment induced by fluoride. The function of DKK1 in the process of this disorder is unknown. Scientifically, we also need to calmly consider few limitations in this study. Firstly, the samples were chosen from one province in China and more high fluoride drinking water areas should be involved in the future study. Secondly, we could not clear which specific aspect of the cognition, such as memory, language, calculations, orientation, attention and concentration, executive functions, visuoconstructional skills, and conceptual thinking, was affected by fluoride via the disfunction of DKK1 and more researches should be performed in the future.

## Conclusions

In conclusion, cognitive impairment could be induced by high fluoride drinking water, and DKK1 may be involved in this process. However, the specific mechanisms are unclear. More studies are needed to determine the function of DKK1, which may as a potential intervention point of this brain damage process that need attention.

## Data Availability

The datasets used or analysed during the current study are available from the corresponding author on reasonable request.

## Abbreviations

AD: Alzheimer’s Disease
APOE: Apolipoprotein E
DKK1: Dickkopf-1
GSH: Glutathione
IQ: Intelligence quotient
MDA: Malondialdehyde
MoCA-B: Montreal Cognitive Assessment-Basic
SOD: Superoxide dismutase
